# Therapeutic Potential of Nanoscale Metal–Organic Frameworks in Hepatocellular Carcinoma

**DOI:** 10.3390/nano15231771

**Published:** 2025-11-26

**Authors:** Helda Tutunchi, Hafezeh Nabipour, Sohrab Rohani

**Affiliations:** 1 Endocrine Research Center, Tabriz University of Medical Sciences, Tabriz 51666-16659, Iran; htoutoun@uwo.ca; 2 Department of Chemical and Biochemical Engineering, University of Western Ontario, London, ON N6A 5B9, Canada

**Keywords:** hepatocellular carcinoma, metal-organic frameworks, drug delivery systems, photodynamic therapy, photothermal therapy, chemodynamic therapy, sonodynamic therapy

## Abstract

Hepatocellular carcinoma (HCC) represents the predominant type of primary liver cancer and remains a major global health concern. Current therapeutic strategies—such as surgical resection, radiation, and chemotherapy—provide clinical benefits but are frequently accompanied by considerable adverse effects. Consequently, identifying alternative treatment modalities and developing strategies that allow the use of lower drug doses without compromising therapeutic outcomes are essential goals in HCC management. Among emerging nanoscale platforms, metal–organic frameworks (MOFs) have attracted exceptional interest as promising candidates for targeted drug delivery in cancer therapy. Their inherent characteristics, including highly ordered porosity, large surface area, tunable cavities, adjustable chemical functionality, and remarkable drug-loading capacity, set them apart from conventional porous nanomaterials. Owing to their hierarchical architecture, MOFs are especially suitable for multimodal and synergistic anti-cancer treatments. MOF-based systems have demonstrated the ability to reinforce the performance of several therapeutic modalities, including photodynamic therapy (PDT), photothermal therapy (PTT), chemodynamic therapy (CDT), and sonodynamic therapy (SDT), while also serving as efficient carriers for targeted drug release. Their structural versatility further enables improved drug stability, enhanced solubility, and controlled-release behavior. This review provides an overview of recent progress in MOF-enabled therapeutic strategies and discusses their potential applications in the treatment of HCC.

## 1. Introduction

The World Health Organization identifies cancer as one of the most serious global threats, with profound impacts on health systems, societies, and economies. It accounts for approximately 16.8% of all deaths and nearly one-quarter of mortality attributed to noncommunicable diseases worldwide [1]. Among various malignancies, liver cancer ranks sixth in incidence and third in cancer-related mortality, placing a significant burden on healthcare resources across the world [2]. Projections indicate that, if current patterns persist, annual liver cancer diagnoses will rise dramatically—from 0.87 million reported in 2022 to an estimated 1.52 million by 2050.

Hepatocellular carcinoma (HCC), the predominant subtype of primary liver cancer, comprises nearly 80% of cases globally. In 2020 alone, over 900,000 new diagnoses were recorded, corresponding to an age-standardized incidence rate (ASIR) of 7.3 per 100,000 individuals [3,4]. Epidemiological data consistently reveal striking regional disparities in HCC occurrence, with particularly high incidence levels observed in East Asia and sub-Saharan Africa, while lower—yet steadily increasing—rates are reported in North America and much of Europe. These geographic differences largely stem from the complex, multifactorial origins of HCC, especially varying prevalence of key etiological factors such as chronic hepatitis B virus (HBV) and hepatitis C virus (HCV) infections, which significantly shape regional incidence trends [4].

Standard therapeutic options for HCC primarily rely on surgical intervention, radiotherapy, and chemotherapy. While conventional anti-cancer agents can exhibit therapeutic benefits, their use is frequently limited by severe adverse effects, largely because cytotoxic drugs are unable to selectively target malignant cells and inevitably damage surrounding healthy tissues [5,6]. Moreover, early-stage HCC often progresses without noticeable symptoms, resulting in many individuals being diagnosed only after the disease has reached an advanced or unresectable stage, at which point curative procedures such as surgery or liver transplantation are no longer viable. For patients with advanced HCC, treatments such as chemotherapy and radiotherapy may help prolong survival and improve overall quality of life; however, their clinical performance is often hindered by poor tumor responsiveness, intrinsic or acquired drug resistance, and diminished radiotherapeutic sensitivity. Furthermore, the need for high therapeutic doses frequently causes substantial toxicity, which can reduce patient adherence to treatment and worsen clinical outcomes [7,8,9]. As a result, there is an urgent need to explore innovative therapeutic modalities and to develop strategies that allow effective treatment at lower drug doses without compromising efficacy [10,11,12].

In recent years, drug delivery systems (DDSs) have attracted significant interest because of their ability to enhance cancer therapy through controlled drug release, site-specific delivery, and improved overall treatment performance [13,14,15]. Advances in nanoscience and nanomedicine have further accelerated the development of innovative therapeutic platforms for cancer management [16,17,18]. A wide spectrum of nanomaterials has been evaluated for targeted drug administration, ranging from inorganic systems—such as mesoporous silica, graphene derivatives, iron oxide nanoparticles (NPs), and quantum dots—to organic carriers like liposomes and polymeric micelles, as well as hybrid organic–inorganic constructs that merge the strengths of both material classes [19,20,21]. Among these technologies, micro- and nanohydrogels have become particularly attractive candidates for drug delivery owing to their customizable porosity, excellent biocompatibility, high water retention capacity, and sensitivity to physiological stimuli including pH, temperature, and enzymatic activity. These features enable them to deliver therapeutic agents in a sustained, localized, and stimulus-responsive manner during cancer treatment [22,23,24,25,26,27,28,29]. Hydrogels are capable of encapsulating a broad range of drugs—whether hydrophilic or hydrophobic—shielding them from premature degradation and providing efficient loading and controlled-release behavior [30,31,32,33]. Their adaptable structure also allows incorporation with diverse components such as polymers, NPs, or metal–organic frameworks (MOFs), thereby improving therapeutic outcomes and enabling multifunctional treatment modalities [34,35,36]. Despite these benefits, the advancement of nanomaterial-based DDSs still faces several barriers. Organic nanocarriers may struggle to achieve highly precise, predictable release profiles and can lack the distinctive physicochemical characteristics needed for multifunctional cancer therapy. In contrast, inorganic materials often exhibit limited biodegradability, restricted chemical tunability, and potential biocompatibility concerns. Therefore, selecting an optimal material platform remains one of the central challenges in the design of effective drug delivery systems [37,38,39,40].

MOFs have recently emerged as one of the most promising classes of nanomaterials for targeted drug delivery in cancer therapy, largely due to their exceptional structural features. MOFs are crystalline porous materials composed of metal ions or metal-containing clusters (such as Fe, Mn, Co, Al, Cu, or Zn) connected to organic linkers—including carboxylate, phosphonate, borate, tetrazolate, sulfonate, or imidazolate groups—forming one-, two- or three-dimensional architectures through coordination bonds [41,42,43,44,45,46]. Often referred to as porous coordination polymers (PCPs), MOFs have attracted considerable interest in both fundamental research and practical engineering applications because of their highly ordered structures and versatile chemical properties. These frameworks have been applied across numerous fields, such as gas storage and separation, water splitting, water treatment, catalysis, sensing, energy-related technologies, bioimaging, and targeted therapeutic delivery [47,48]. Their hybrid organic–inorganic nature provides a combination of advantages, including biodegradability, biocompatibility, and safety, which are crucial for biomedical use. Furthermore, MOFs offer exceptionally high porosity, large surface areas, adjustable pore dimensions, and modifiable chemical functionalities, making them ideal candidates for high-capacity drug loading and controlled-release applications [49,50].

Compared with conventional porous nanoparticles, the hierarchical architecture of metal–organic frameworks (MOFs) has been shown to significantly improve their performance in multimodal anti-cancer therapies. As a result, MOFs have become central to the design of advanced tumor-targeted drug delivery systems [50,51,52]. These materials can substantially enhance the effectiveness of various therapeutic strategies, including photodynamic therapy (PDT), photothermal therapy (PTT), chemodynamic therapy (CDT), sonodynamic therapy (SDT), as well as precise drug delivery [53,54,55,56]. The tunable structure of MOFs also makes them highly suitable as drug carriers, offering abundant binding sites, increased stability, enhanced solubility, and the ability to release therapeutic agents in a controlled manner [57,58,59,60]. Additionally, MOFs can transport a wide range of therapeutic molecules—including chemotherapeutic drugs, photosensitizers, and radiosensitizers—directly to tumor tissues, thereby improving treatment outcomes while minimizing unintended side effects on healthy cells [61,62,63,64,65].

## 2. Metal–Organic Frameworks-Based Therapies for Hepatocellular Carcinoma

With their precisely defined architectures, MOFs can induce tumor cell death through SDT, PDT, PTT, or CDT. This section highlights the therapeutic potential of MOFs in HCC, with a particular focus on PDT, PTT, CDT, and SDT.

### 2.1. Metal–Organic Frameworks for Photodynamic Therapy of Cancer

PDT, which utilizes photosensitizers (PSs) activated by light at specific wavelengths to produce reactive oxygen species (ROS), has recently gained attention as a non-invasive alternative to traditional cancer treatments. PDT is regarded as a precise and safe therapeutic approach, as it selectively targets malignant cells while minimizing damage to healthy tissues [66,67]. In MOF-mediated PDT, PS-loaded MOFs—such as porphyrin-based frameworks—are directed to tumor sites and subsequently exposed to light of the appropriate wavelength to activate the photosensitizer. The activated PS does not directly attack biomolecules; instead, it transfers energy from light to molecular oxygen, generating ROS such as singlet oxygen (^1^O_2_), superoxide radicals (O_2_^−•^), hydroxyl radicals (HO^•^), and hydrogen peroxide (H_2_O_2_), which induce tumor cell death and tissue destruction [68]. Unlike chemotherapy or radiotherapy, which can cause systemic toxicity, the ROS produced during PDT are confined to the target site and do not exert harmful effects on the rest of the body. Due to these advantages—non-invasiveness, localized activity, and minimal side effects—PDT has been widely employed for treating superficial tumors [68,69].

Liu et al. [70] synthesized nanoscale porphyrin-based MOFs (NPMOFs) by combining biocompatible zirconium ions (Zr^4+^) with meso-tetrakis(4-carboxyl)-21H,23H-porphine (TCPP) using a microemulsion approach. Doxorubicin (DOX) was employed as a model chemotherapeutic agent to evaluate both its loading capacity and pH-responsive release from the NPMOFs. In vitro studies using HepG2 liver cancer cells demonstrated that the extensive pore network of NPMOFs enabled DOX encapsulation levels up to 109%. When combined with 655 nm laser irradiation, DOX@NPMOFs exhibited a low half-maximal inhibitory concentration (IC_50_ = 67.72 µg mL^−1^) and induced approximately 90% cell death. The in vivo efficacy of the combined chemotherapy and PDT approach was further investigated in HepG2 tumor-bearing mice. Fluorescence imaging was used to monitor NPMOF accumulation at tumor sites, and once maximal localization was achieved, tumors in PDT groups were irradiated with a 655 nm laser (180 J cm^−2^ for 15 min). Tumor progression was then compared across groups receiving monotherapy, combination therapy, or saline control. While tumor growth was partially suppressed in groups treated with either chemotherapy or PDT alone, mice receiving DOX@NPMOFs co-therapy exhibited marked tumor regression within two days. Notably, two tumors were completely eradicated, while the remaining two shrank from 62.5 mm^3^ to 2 mm^3^ over 10 days. These findings indicate that DOX@NPMOFs were efficiently internalized by cancer cells and exerted potent cytotoxic effects when applied in combination therapy. Importantly, no observable damage to surrounding skin or tissues was reported following PDT treatment.

**Figure 1:**
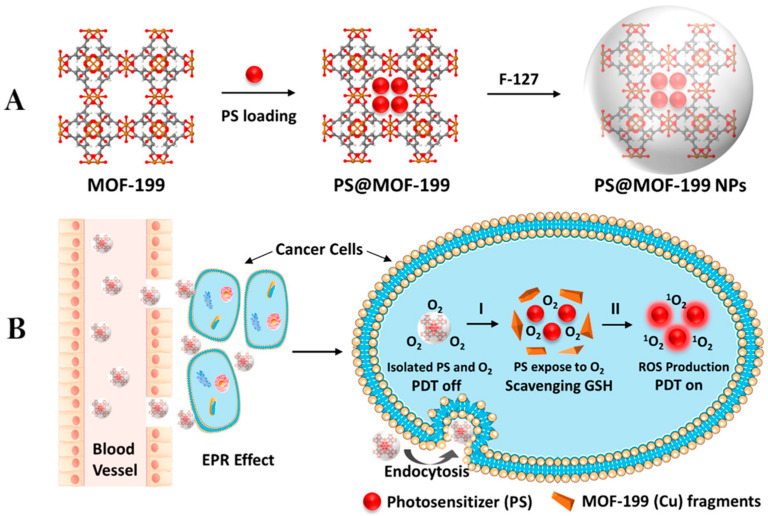
(**A**) Stepwise synthesis of PS@MOF-199 and F127-coated PS@MOF-199 nanoparticles (PS@MOF-199 NPs). (**B**) Illustration of the photosensitization quenching and activation behavior of PS@MOF-199 NPs within the tumor microenvironment. Process I: Framework collapse of MOF-199 initiated by intracellular GSH. Process II: ROS production under light irradiation. Reproduced with permission from ref. [71] (copyright 2019, Wang).

As illustrated in Figure 1A, Wang et al. [71] developed a multifunctional MOF-based nanoplatform (PS@MOF-199 NPs) for image-guided PDT. They first synthesized MOF-199, a Cu(II) carboxylate-based framework, and incorporated two PSs—a commercial one, chlorin e6 (Ce6), and a synthesized aggregation-induced emission (AIE) compound, 2-[4-(dimethylamino)phenyl]anthracene-9,10-dione (TPAAQ)—via physical adsorption to obtain PS@MOF-199. To improve dispersibility and biocompatibility, the composite was further encapsulated with polyether F127 through a thin-film dispersion process, yielding PS@MOF-199 NPs. As shown in Figure 1B, the nanoplatform remains photosensitization-inactive when outside cells, since the intact MOF-199 framework restricts oxygen access to the encapsulated PSs. After cellular internalization through the enhanced permeability and retention (EPR) effect, intracellular glutathione (GSH) induces the degradation of MOF-199, releasing TPAAQ or Ce6 to react with O_2_ and generate ^1^O_2_ under light irradiation for PDT. Simultaneously, Cu^2+^ ions released from the decomposed MOF deplete endogenous GSH through redox reactions, thereby amplifying the ROS-mediated cytotoxicity. The resulting PS@MOF-199 NPs displayed potent phototoxicity toward HepG2 cells and significant tumor suppression in a transgenic zebrafish liver tumor model following irradiation. This approach effectively integrates AIE and conventional PSs within a degradable MOF carrier, achieving oxygen-responsive, image-guided PDT with enhanced therapeutic efficacy and minimal nonspecific phototoxicity.

Cai et al. [72] developed photosensitizer-loaded MOF nanoparticles (PS-MOF NPs) designed to eliminate residual tumor cells following PDT by combining PDT with antihypoxic modulation and immunostimulatory agents, effectively functioning as an in situ tumor vaccine to boost long-term anti-tumor immune responses. The MOF NPs, referred to as PCN224 (PCN), were self-assembled from H_2_TCPP (photosensitizer) and zirconium ions through coordination interactions, incorporating a hypoxia-inducible factor (HIF) signaling inhibitor (ACF) and the immunologic adjuvant CpG, with a hyaluronic acid (HA) coating applied to the surface (PCN-ACF-CpG@HA). The PCN component within the NPs demonstrated strong PDT-mediated cytotoxicity against tumor cells. Meanwhile, ACF effectively suppressed HIF-1α-driven survival and metastatic pathways that are typically activated post-PDT. Importantly, the combination of CpG with tumor-associated antigens (TAAs) released from damaged tumor cells acted as a localized tumor vaccine, further stimulating the host’s immune system to target and eliminate residual malignant cells. Collectively, this integrated approach represents a promising strategy to synergistically enhance PDT outcomes while simultaneously invoking anti-tumor immunity.

Fu et al. [46] developed a MOF-based nanocomposite incorporating Ce6 to enhance its PDT performance under physiological conditions. Although Ce6 is a widely used second-generation photosensitizer, its clinical utility is limited by poor water solubility, tendency to aggregate in biological media, and insufficient accumulation in tumor tissues, which reduce its therapeutic efficiency. To address these limitations, the researchers employed an appropriate drug delivery carrier. They designed a therapeutic agent by incorporating Ce6 into a zeolitic imidazolate framework-8 (ZIF-8) modified with hyaluronic acid (HA), creating the ZIF-8@Ce6–HA nanocomposite via a one-pot self-assembly approach. ZIF-8, a subclass of MOFs composed of zinc ions coordinated with 2-methylimidazole, is well-suited for targeted drug delivery because it can degrade in the acidic tumor microenvironment. The strong electrostatic interactions between Zn^2+^ ions and Ce6 molecules enabled high loading efficiency of Ce6 within the MOF structure. Surface modification with HA improved the biocompatibility of the nanocarrier and reduced systemic toxicity. Characterization revealed that ZIF-8@Ce6–HA exhibited high encapsulation efficiency, effective cellular uptake, and excellent biocompatibility. Mass spectrometry analysis indicated that HA modification prolonged blood circulation time and minimized systemic side effects. In vitro studies with HepG2 liver cancer cells showed that free Ce6 caused only about 29.5% cell death following irradiation due to ROS generation, limited by aggregation in aqueous media. In contrast, ZIF-8@Ce6–HA significantly enhanced PDT efficacy, resulting in approximately 88.4% cell death after irradiation. These results demonstrate that ZIF-8@Ce6–HA is a promising and effective platform for photodynamic therapy.

Hu et al. [47] engineered a targeted, synergistic therapy combining PDT and chemotherapy using a glyco-modified MOF nanostructure, termed DOX@Gal-PCN-224, administered via percutaneous transperitoneal puncture, to treat HCC in a mouse model of in situ liver tumors. The chemotherapeutic agent DOX was incorporated into the MOF during a one-pot synthesis, enhancing drug loading and enabling pH-responsive release. Furthermore, the cytotoxic effect of DOX was amplified by local hypoxia resulting from PDT-induced oxygen consumption. The NPs were functionalized with galactose to selectively target the asialoglycoprotein receptor (ASGPR), which is highly expressed on hepatocytes. This targeting capability was confirmed in HepG2 and Huh7 cell lines, demonstrating reduced off-target toxicity in healthy tissues. The intrinsic fluorescence of both DOX and the photosensitizer allowed real-time imaging to monitor NP distribution in both subcutaneous and orthotopic tumor models, confirming effective liver-specific delivery. Following a single interventional PDT irradiation, tumor growth in orthotopic HCC-bearing mice was suppressed by approximately 98%, highlighting the strong potential of this combined interventional PDT and chemotherapy strategy for future clinical applications in HCC treatment.

Pan et al. [48] developed a bimetallic MOF nanoplatform, ZMRPC@HA, containing ruthenium (Ru^3+^) and platinum (Pt^4+^) ions, designed to modulate the tumor microenvironment and enhance PDT for hypoxic tumors. The MOFs were loaded with the photosensitizer Ce6 and surface-modified with HA to improve biocompatibility and tumor targeting. ZMRPC@HA exhibited catalase- and glutathione oxidase-like activities, primarily due to Ru^3+^, enabling the generation of oxygen and depletion of glutathione within the tumor, thereby synergistically improving PDT efficacy under hypoxic conditions. The PDT performance of ZMRPC@HA was first evaluated in vitro using Hepatoma 22 (H22) cells, demonstrating significant inhibition of cellular proliferation. Subsequent in vivo studies in BALB/c mice bearing H22 tumor xenografts confirmed that ZMRPC@HA effectively suppressed tumor growth under 660 nm laser irradiation. Both in vitro and in vivo results indicated that this MOF-based PDT approach could efficiently hinder tumor cell differentiation and proliferation even in deep tissue regions, highlighting its potential as a potent strategy for treating hypoxic tumors.

### 2.2. Metal–Organic Frameworks for Photothermal Therapy of Cancer

PTT is recognized for its non-invasive nature, localized therapeutic effects, high efficiency, and minimal side effects. In PTT, specific MOFs absorb near-infrared (NIR) light and convert it into heat, generating localized hyperthermia that selectively destroys tumor cells. Essentially, PTT can be considered an extension of photodynamic therapy (PDT), where a photosensitizer absorbs light within a specific wavelength range and transforms optical energy into thermal energy, allowing precise ablation of malignant cells while sparing adjacent healthy tissues [49,50]. Furthermore, combining PDT and PTT has been shown to produce synergistic antitumor effects with reduced side effects. Heat generated during PTT can improve local blood circulation, thereby increasing oxygen availability in tumor tissues and enhancing the efficacy of oxygen-dependent PDT. Simultaneously, ROS produced during PDT can disrupt tumor physiology and alter the microenvironment, which further sensitizes cancer cells to hyperthermia and improves the overall therapeutic outcome [51,52,53].

Shi and colleagues [54] developed a novel theranostic nanoparticle system (MINPs) through a straightforward self-assembly approach, incorporating manganese ions (Mn^2+^) and indocyanine green (ICG), stabilized by poly(vinylpyrrolidone) (PVP). In vitro studies demonstrated that MINPs significantly inhibited the proliferation of HepG2 liver cancer cells when exposed to 808 nm laser irradiation. For in vivo evaluation, MINPs were administered to mice bearing subcutaneous tumors, and the photoacoustic (PA) signal around the tumor site was monitored for 12 h. The PA signal in the MINP-treated group was approximately three times higher than that in the control group receiving free ICG, indicating enhanced accumulation. Similarly, magnetic resonance imaging (MRI) revealed amplified positive tumor signals over time, with quantitative analysis showing that MRI intensity at 12 h post-injection was 1.8 times greater than pre-injection levels. Both in vitro PTT experiments and in vivo imaging confirmed that MINP treatment induced significant necrosis and tumor cell damage in HepG2 tumors, whereas control groups displayed negligible effects. These findings suggest that MINPs represent a promising platform for multimodal imaging-guided PTT of hepatocellular carcinoma, offering potential for future clinical applications.

Zhou et al. [34] developed HCC-targeted NPs by conjugating the SP94 peptide and cyanine 5.5 (Cy5.5) to Prussian blue (PB) NPs, which were loaded with the chemotherapeutic drug sorafenib (SF), forming SP94-PB-SF-Cy5.5 NPs. These FDA-approved nanoparticles were designed for HCC-specific multimodal imaging and combined PTT with targeted chemotherapy. The researchers tested the NPs in both human (HepG2) and mouse (Hepa1–6) HCC cell lines and assessed their imaging and therapeutic efficacy in vitro and in vivo. The SP94-PB-SF-Cy5.5 NPs accumulated preferentially at HCC tumor sites and were suitable for near-infrared fluorescence (NIRF), photoacoustic imaging (PAI), and MRI, enabling triple-modality imaging. The NPs exhibited strong photothermal effects and facilitated controlled release of SF, effectively eradicating tumors with minimal local recurrence and low systemic toxicity. Additionally, the catalase-like activity of PB enhanced oxygen generation, alleviating tumor hypoxia. The combination of PTT and hypoxia mitigation synergistically promoted an immune-stimulating tumor microenvironment. When combined with anti-PD-L1 monoclonal antibody therapy, SP94-PB-SF-Cy5.5 NPs under NIR irradiation produced notable abscopal effects, significantly reducing metastasis and tumor recurrence. This combinational approach also induced long-term antitumor immune memory, preventing tumor regrowth. Overall, these multifunctional and safe NPs offer a promising strategy for effective HCC treatment.

### 2.3. Metal–Organic Frameworks for Chemodynamic Therapy of Cancer

CDT is an emerging cancer treatment approach that utilizes CDT agents to convert H_2_O_2_ into highly reactive HO^•^ via Fenton or Fenton-like reactions, thereby inducing tumor cell apoptosis and necrosis. In MOF-catalyzed Fenton reactions, Fe^2+^ ions within the MOF facilitate the transformation of H_2_O_2_ into HO^•^, causing oxidative damage specifically in cancer cells. Additionally, other metal ions such as Cu^+^, Mn^2+^, Co^2+^, and Ti^3+^ have been reported to mediate similar Fenton-like reactions, generating HO^•^ from H_2_O_2_ [73]. Combining CDT with PDT can produce synergistic antitumor effects. The oxygen generated during CDT can enhance the effectiveness of PDT, while the potent cytotoxicity of HO^•^ directly kills tumor cells, further amplifying tumor suppression. Recent research has systematically explored this combination, demonstrating that the concurrent application of PDT and CDT increases oxidative stress in tumors and achieves superior therapeutic outcomes compared to either monotherapy alone [74,75].

Ding et al. [76] synthesized iron-based MOF nanoparticles (FeMOFs NPs) using TCPP (Fe) and zirconium clusters as structural units. The hydrophobic chemotherapeutic agent camptothecin (CPT) was incorporated, and small gold (Au) NPs were deposited on the surface in situ, resulting in the novel PEG-Au/FeMOF@CPT NPs. The surface Au NPs were further functionalized with 1-dodecanethiol (C12SH) and methoxy polyethylene glycol thiol (PEG-SH) to enhance stability and biocompatibility. The antitumor activity of CDT was assessed in HepG2 cells using the MTT (3-(4,5-dimethylthiazole-2-yl)-2,5-diphenyl tetrazolium bromide) assay. For CPT monotherapy, the IC_50_ was 206 ± 22 × 10^−9^ M. Treatment with PEG-Au/FeMOF NPs, combining cascade starvation and CDT, significantly inhibited cell proliferation with an IC_50_ of 3.51 ± 0.26 µg mL^−1^. The combination of chemotherapy and CDT using PEG-Au/FeMOF@CPT NPs yielded the lowest IC_50_ value at 0.31 ± 0.04 µg mL^−1^. These findings indicate a strong synergistic effect between CDT and CPT, effectively enhancing tumor growth suppression.

### 2.4. Metal–Organic Frameworks for Sonodynamic Therapy of Cancer

SDT is an emerging ultrasound-based therapeutic strategy that generates ROS through acoustic cavitation and sonochemical reactions. Compared with PDT, SDT offers deeper tissue penetration, non-invasive tumor ablation, and high therapeutic precision, making it particularly suitable for HCC. However, its efficacy is often limited by insufficient ROS production and tumor hypoxia. The incorporation of MOFs as sonosensitizers or catalytic carriers has shown significant potential in overcoming these limitations and improving therapeutic outcomes [77,78,79,80,81,82].

Porphyrinic MOFs such as Mn–porphyrin and Cu–TCPP frameworks possess intrinsic sonosensitizing properties due to their conjugated ligands and redox-active metal centers, enabling efficient ultrasound-triggered ROS generation. In HepG2 and H22 hepatoma models, these nanosystems markedly enhanced oxidative stress and mitochondrial damage, resulting in significant antitumor effects [80,83,84]. Similarly, ZIF-8- and Fe-MOF-based nanocomposites loaded with Ce6 or hematoporphyrin have exhibited pH-responsive degradation and strong ROS formation under ultrasound, achieving high cytotoxicity against HCC cells in vitro and tumor suppression in vivo [85,86].

Some MOF platforms designed for PDT, including DOX@Gal-PCN-224 and ZMRPC@HA, have also shown efficient ultrasound-induced ROS generation and oxygen-regulating activity, indicating their potential as dual PDT/SDT nanoplatforms for liver cancer [47,48,87]. Overall, MOF-based nanosonosensitizers integrate oxygen generation, GSH depletion, and ROS amplification, offering a versatile and biocompatible platform for precise, ultrasound-activated therapy of HCC [88,89].

## 3. Conclusions and Future Directions

The design of functional MOFs for cancer phototherapy has emerged as a rapidly advancing area of research. These phototherapeutic MOFs can be constructed directly by coordinating PSs or photothermal agents with metal ions or clusters. In addition, their tunable pore sizes make MOFs excellent carriers for phototherapeutic agents. The highly ordered frameworks prevent aggregation of PSs and photothermal agents, thereby markedly improving their therapeutic efficiency. MOFs can also be easily functionalized with small molecules, biomacromolecules, or other active materials, enabling targeted delivery, imaging-guided treatment, and synergistic therapeutic strategies [90,91,92]. Nevertheless, despite these advantages, significant challenges must still be addressed before MOFs can advance to clinical trials.

(1) Currently available phototherapeutic agents face notable limitations, such as restricted tissue penetration of light used in phototherapy, dependence on oxygen for ROS generation in PDT, and non-uniform heat distribution in PTT. Although strategies like upconversion NPs or two-photon-activated photosensitizers can enhance light penetration, targeting deep organs like the liver remains difficult. As a result, imaging-guided approaches have become a critical component in phototherapy. Minimally invasive laparoscopic techniques allow optical fibers to access deeper tissues, activating photosensitizers for effective treatment. Such methods have already advanced to clinical applications and show great promise for precision cancer theranostics. Additionally, light-independent modalities, including chemiluminescence and bioluminescence, offer alternative means to overcome the limitations associated with light penetration [50,93,94].
(2) To improve the biosafety of MOFs as drug carriers, it is important to use biocompatible ligands and metal ions, such as Fe, Ca, and Zn. Surface modifications using peptides, antibodies, or small molecules have proven effective for enhancing tumor targeting and accumulation. Despite these advances, the pharmacokinetics of many MOF systems remain inadequately studied. Once MOFs reach the tumor site, they should degrade efficiently into small molecules or ions that can be metabolized safely, minimizing adverse effects. Although various stimuli—including pH, biothiols, ATP, and hypoxic conditions—have been investigated to trigger MOF degradation, their effectiveness requires further optimization [95,96,97,98,99,100,101].

In summary, precisely engineered and functional MOFs have demonstrated potential for PDT, PTT, SDT, and CDT in cancer therapy [102]. Table 1 summarizes key MOF-based nanosystems for PDT, PTT, and CDT in hepatocellular carcinoma. While this research area is still emerging, ongoing developments are expected to produce increasingly sophisticated MOFs suitable for clinical translation.

## Figures and Tables

**Table 1:** Representative MOF-based nanosystems for PDT, PTT, and CDT therapies in hepatocellular carcinoma.

Study (Ref)	MOF Type/NP	Payload	Therapy Type	In Vitro IC_50_/Efficacy	In Vivo Model	Main Outcomes
Liu et al. [70]	Zr-TCPP NPMOF	DOX	PDT + Chemo	67.72 µg mL^−1^; 90% cell lethality	HepG2 tumor-bearing mice	Two tumors disappeared, two shrank from 62.5 → 2 mm^3^; no skin/tissue damage
Wang et al. [71]	MOF-199	Ce6	PDT	High phototoxicity to HepG2	Zebrafish larvae	GSH-responsive PS release; tumor volume reduced; improved imaging-guided PDT
Cai et al. [72]	PCN-224@ACF-CpG@HA	Ce6 + ACF + CpG	PDT + Immunotherapy	—	Tumor-bearing mice	Eliminated residual tumor cells; activated host anti-tumor immune response
Fu et al. [46]	ZIF-8@Ce6–HA	Ce6	PDT	88.4% HepG2 cell death	HepG2 cells	Enhanced Ce6 solubility, efficient uptake, mitigated aggregation; improved PDT efficacy
Hu et al. [47]	Gal-PCN-224	DOX + Ce6	PDT + Chemo	—	Orthotopic HCC mouse model	Tumor growth inhibited by 98%; ASGPR-targeted delivery; imaging-guided therapy
Pan et al. [48]	ZMRPC@HA	Ce6	PDT	—	H22 tumor xenograft, BALB/c mice	Synergistic ROS and O_2_ production; effective inhibition under 660 nm laser
Shi et al. [54]	Mn^2+^–ICG MINPs	ICG	PTT	Significant HepG2 inhibition under 808 nm laser	Subcutaneous HepG2 tumors in mice	Tumor necrosis; enhanced MRI and PA imaging; time-dependent accumulation
Zhou et al. [34]	SP94-PB-SF-Cy5.5 NPs	Sorafenib	PTT + Targeted therapy	Effective tumor ablation	HepG2 and Hepa1–6 mouse models	High HCC accumulation; triple-modality imaging; hypoxia relief; immune activation; prevented recurrence
Ding et al. [76]	PEG-Au/FeMOF@CPT	CPT	CDT + Chemo	0.31 ± 0.04 µg mL^−1^	HepG2 cells	Strong synergistic tumor suppression: CDT enhanced CPT cytotoxicity

## Data Availability

No new data were created or analyzed in this study.

## Abbreviations

ACF, hypoxia-inducible factor (HIF) signaling inhibitor; ACQ, aggregation-caused quenching; AIE, aggregation-induced emission; ASGPR, asialoglycoprotein receptor; ASIR, age-standardized incidence rate; ATP, adenosine triphosphate; Au, gold; C12SH, 1-dodecanethiol; CDT, chemodynamic therapy; Ce6, chlorin e6; CPT, camptothecin; Cy, cyanine; DDSs, drug delivery systems; DOX, doxorubicin; EPR, enhanced permeability and retention; Fe, iron; FeMOF, iron-based metal–organic framework; F127, polyether F127; GSH, glutathione; HA, hyaluronic acid; HBV, hepatitis B virus; HCC, hepatocellular carcinoma; HCV, hepatitis C virus; HIF, hypoxia-inducible factor; HO^•^, hydroxyl radical; H_2_O_2_, hydrogen peroxide; IC_50_, half-maximal inhibitory concentration; ICG, indocyanine green; IGTSs, imaging-guided therapy systems; MRI, magnetic resonance imaging; MINPs, manganese-based indocyanine green nanoparticles; MOF, metal–organic framework; MOFs, metal–organic frameworks; MS, mass spectrometry; NIR, near-infrared; NIRF, near-infrared fluorescence; NP, nanoparticle; NPs, nanoparticles; NPMOFs, nanoscale porphyrinic metal–organic frameworks; PA, photoacoustic; PAI, photoacoustic imaging; PCN, porous coordination network; PEG, polyethylene glycol; PEG-SH, methoxy polyethylene glycol thiol; PDT, photodynamic therapy; PD-L1, programmed death-ligand 1; PHS, photosensitizer; PHS@MOF, photosensitizer-loaded metal–organic framework; PVP, polyvinylpyrrolidone; Pt^4+^, platinum; PTT, photothermal therapy; ROS, reactive oxygen species; Ru^3+^, ruthenium; ^1^O_2_, singlet oxygen; SF, sorafenib; SDT, sonodynamic therapy; SP94, HCC-targeting peptide SP94; O_2_^−•^, superoxide radicals, TAAs, tumor-associated antigens; TCPP, meso-tetrakis(4-carboxyl)-21H,23H-porphine; TPAAQ, 2-[4-(dimethylamino)phenyl]anthracene-9,10-dione; Ti^3+^, titanium; ZIF-8, zeolitic imidazolate framework-8; ZMRPC, bimetallic MOF nanoplatform; Zr, zirconium.
